# Inferring the age and sex of ancient potters from fingerprint ridge densities: A data-driven, Bayesian mixture modelling approach

**DOI:** 10.1016/j.mex.2023.102292

**Published:** 2023-07-26

**Authors:** Andrew T. Burchill, Akiva Sanders, Thomas J.H. Morgan

**Affiliations:** a School of Life Sciences, Arizona State University; b Department of Near Eastern Languages and Civilizations, University of Chicago; c American Research Institute in Turkey; d School of Human Evolution and Social Change, Arizona State University; e Institute of Human Origins, Arizona State University

**Keywords:** Mean ridge breadth (MRB), Ridge density (RD), Demography, Dermatoglyphs, Dermatoglyphic Analysis, Ceramics, Ceramic shrinkage, Model comparison, Bayesian dermatoglyphic evaluation

## Abstract

The density of epidermal ridges in a fingerprint varies predictably by age and sex. Archaeologists are therefore interested in using recovered fingerprints to learn about the ancient people who produced them. Recent studies focus on estimating the age and sex of individuals by measuring their fingerprints with one of two similar metrics: mean ridge breadth (MRB) or ridge density (RD). Yet these attempts face several critical problems: expected values for adult females and adolescent males are inherently indistinguishable, and inter-assemblage variation caused by biological and technological differences cannot be easily estimated. Each of these factors greatly decreases the accuracy of predictions based on individual prints, and together they condemn this strategy to relative uselessness. However, information in fingerprints from across an assemblage can be pooled to generate a more accurate depiction of potter demographics. We present a new approach to epidermal ridge density analysis using Bayesian mixture models with the following key benefits:

• Age and sex are estimated more accurately than existing methods by incorporating a data-driven understanding of how demographics and ridge density covary.
• Uncertainty in demographic estimates is automatically quantified and included in output.
• The Bayesian framework can be easily adapted to fit the unique needs of different researchers.

## Graphical abstract

**Figure:**
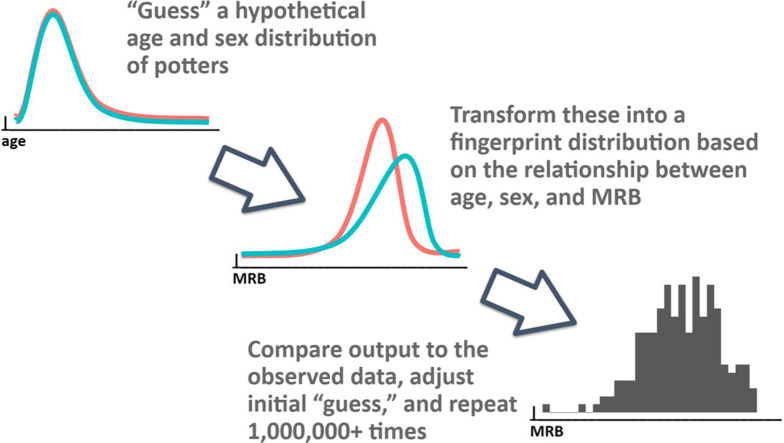

Specifications table

**Table:** 

Subject Area:	Mathematics and statistics
More specific subject area:	*Dermatoglyphics*
Method name:	*Bayesian dermatoglyphic evaluation*
Name and reference of original method:	*N/A*
Resource availability:	*R code available at*https://github.com/andburch/bayesianfingerprintr

## *Method details

### Rationale

Over the past five years, archaeologists have become increasingly interested in using recovered fingerprints impressions to infer the demographics of the potters involved in ceramic production. Studies of modern fingerprints show that the density of epidermal ridges varies predictably with age and sex: women and adolescents have narrower sets of ridge-furrow pairs within their fingerprints than adult men (e.g. [1], [2], [3], [4]). Most recent archaeological studies use this variation in epidermal ridge density to attempt predicting the age and sex of individual ceramic producers.

Yet this method encounters several problems: firstly, because of how epidermal ridge density varies with both sex and age, expected values for adult women and younger adolescent men are effectively indistinguishable. Secondly, adult fingerprint measures differ between populations due to a combination of genetic and lifestyle factors. For instance, a set of fingerprints may resemble those of contemporary Western women, yet nonetheless have been produced by men from a population with lower than typical ridge density, perhaps because of prevalent nutritional deficiencies. Additionally, fingerprints scale and distort after their creation in a process called ceramic shrinkage, the extent of which depends on differences in clay body and firing technique. Each of these factors greatly decreases the accuracy of predictions based on individual prints, and together they pose a serious impediment to the reconstruction of demographic factors from fingerprints.

However, with several reasonable assumptions, and while recognizing the uncertainty introduced by the above challenges, information in fingerprint measurements from across an assemblage can be pooled to create a more accurate depiction of potter demographics. In this paper, we present a new approach to epidermal ridge density analysis using Bayesian mixture models and a more data-driven understanding of how demographics and ridge density covary.

### Measuring fingerprints: mean ridge breadth and ridge density both measure the same quality

Recent archaeological studies measure the density of epidermal ridges on prints using one of two metrics: mean ridge breadth (MRB) or ridge density (RD). MRB is calculated by measuring the distance across any number of sequential ridges from crest to crest and dividing by the number of ridges measured. This metric is used in studies such as [5], Kantner et al. [6] and [7]. RD is measured by counting the discrete number of fingerprint ridges that cross a diagonal line within a 5-by-5 mm square. This metric is used in studies such as Sanders 2015, Taduran et al. [8], Redomero et al. [9], Nayak et al. [2], Sharma et al. [3], Gungadin [10], Acree [1].

**Fig. 1:**
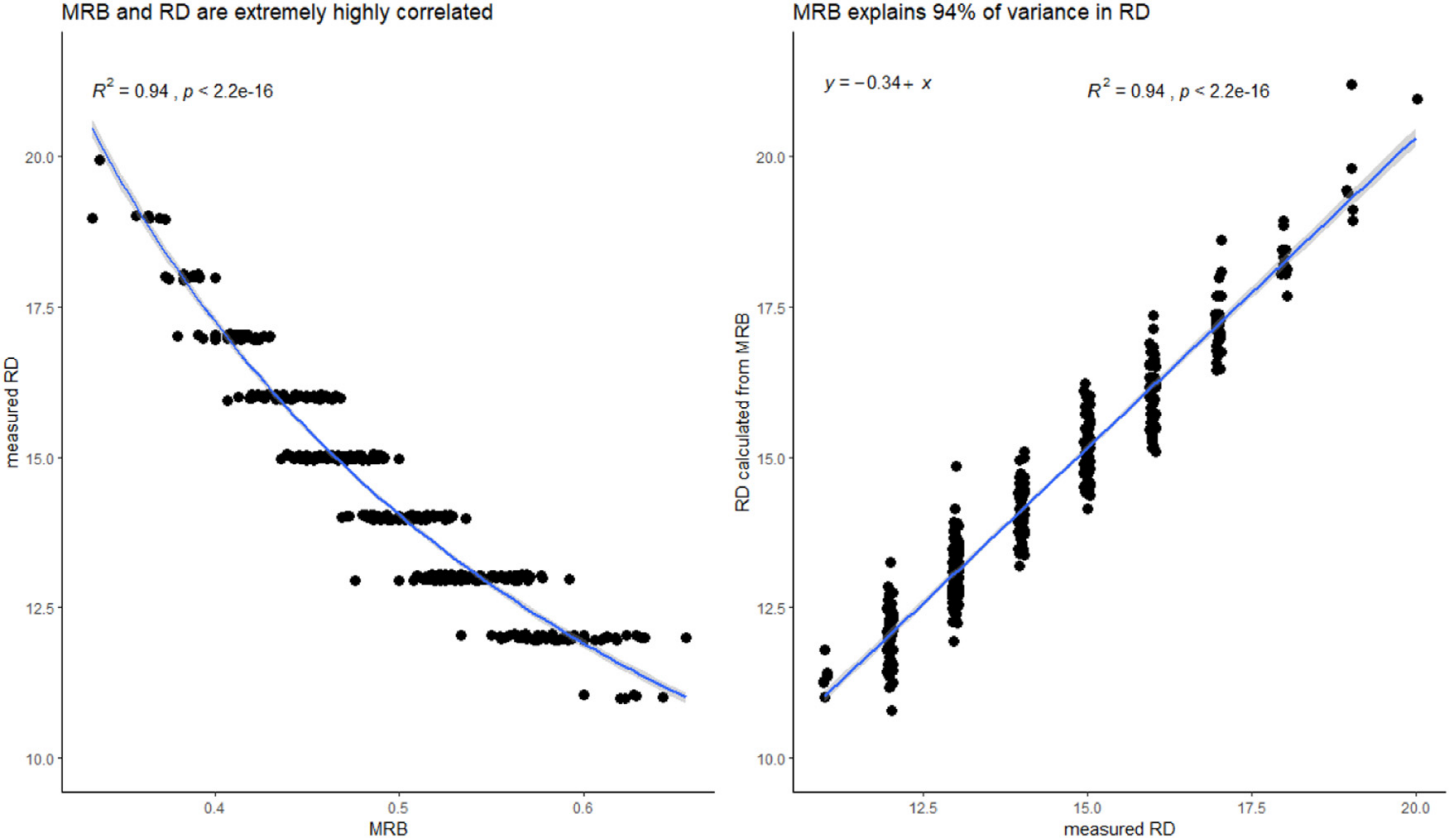
Mean ridge breadth (MRB) and ridge density (RD) metrics essentially measure the same quality. Fingerprints from the site of Hama, Syria were measured using both methods and then compared. As expected, measured MRB and RD values have an inverse relationship and a very high correlation, shown on the left. Also as expected, RD can be calculated from MRB using the equation RD = 5√2 / MRB, shown on the right. Even if one were unaware of the true inverse relationship between these two formulations of epidermal ridge density, they have still a direct linear correlation of -0.96 in our data (not shown).

In other words, RD measures the number of ridges per unit of distance, while MRB measures the distance per number of ridges. These two metrics are conceptual inverses of one another, meaning they are different sampling strategies for the same underlying value. (Note that the ratio of ridge breadth to furrow breadth does not play a role in either calculation). As proof of their equivalence, in our archaeological dataset where both metrics were measured [11], they are clearly inversely related and very highly correlated. Variation in MRB values explains approximately 94% of the variance in RD values (Fig. 1). As a consequence, these two metrics *cannot* be used for different purposes because they are effectively measuring the same thing (contra [12,13]). Additionally, contrary to claims that these metrics measure different types of demographic information (specifically age and sex), recent studies have shown that *both* RD [4,[14], [15], [16]] and MRB [5,17,18] measurements vary predictably with both sex *and* age.

Given that the RD metric involves discretizing a continuous value into integer bins, it suffers from information loss and a decrease in the analytical quality of the data. Therefore, we suggest using MRB as the measure of epidermal ridge density. In the following document, we will primarily focus our attention on MRB statistics over RD, when possible.

### Limitations in using epidermal ridge density to estimate demographics

Although epidermal ridge density does vary with age and sex, there are several hurdles preventing its use in inferring ancient demographics.

#### Problem 1: Adult women and young men are non-identifiable

Imagine the development of a human fingerprint through adulthood. The number and arrangement of fingerprint ridges is determined months before birth [19], and both sexes are born with similar ridge densities. As someone ages, their hands and fingers grow in size, and these existing, fixed ridges are stretched further apart from one another. Thus, the average breadth of ridge-furrow pairs—therefore the average density of ridges—is highly correlated to the height and size of the individual ([7]: 313). Additionally, from two years of age until adolescence, height [20] and average MRB increase near-linearly with an individual's age, and over this time course these trends are not significantly different between sexes [7,5]. Thus, MRB can be a useful tool in estimating the age, but not sex, of pre-adolescent children.

However, female adolescents stop growing approximately two years earlier than males, on average (13-14 vs. 15-16, [20]). Thus, there is a statistical difference between the heights (and MRB values) of adult men and adult women. Once an individual's adult height is reached, epidermal ridge density remains constant, so MRB can be used to estimate the sex of adults, but not their specific ages. Thus, MRB allows the estimate of the age, but not sex of children, and the sex, but not age, of adults.

**Fig. 2:**
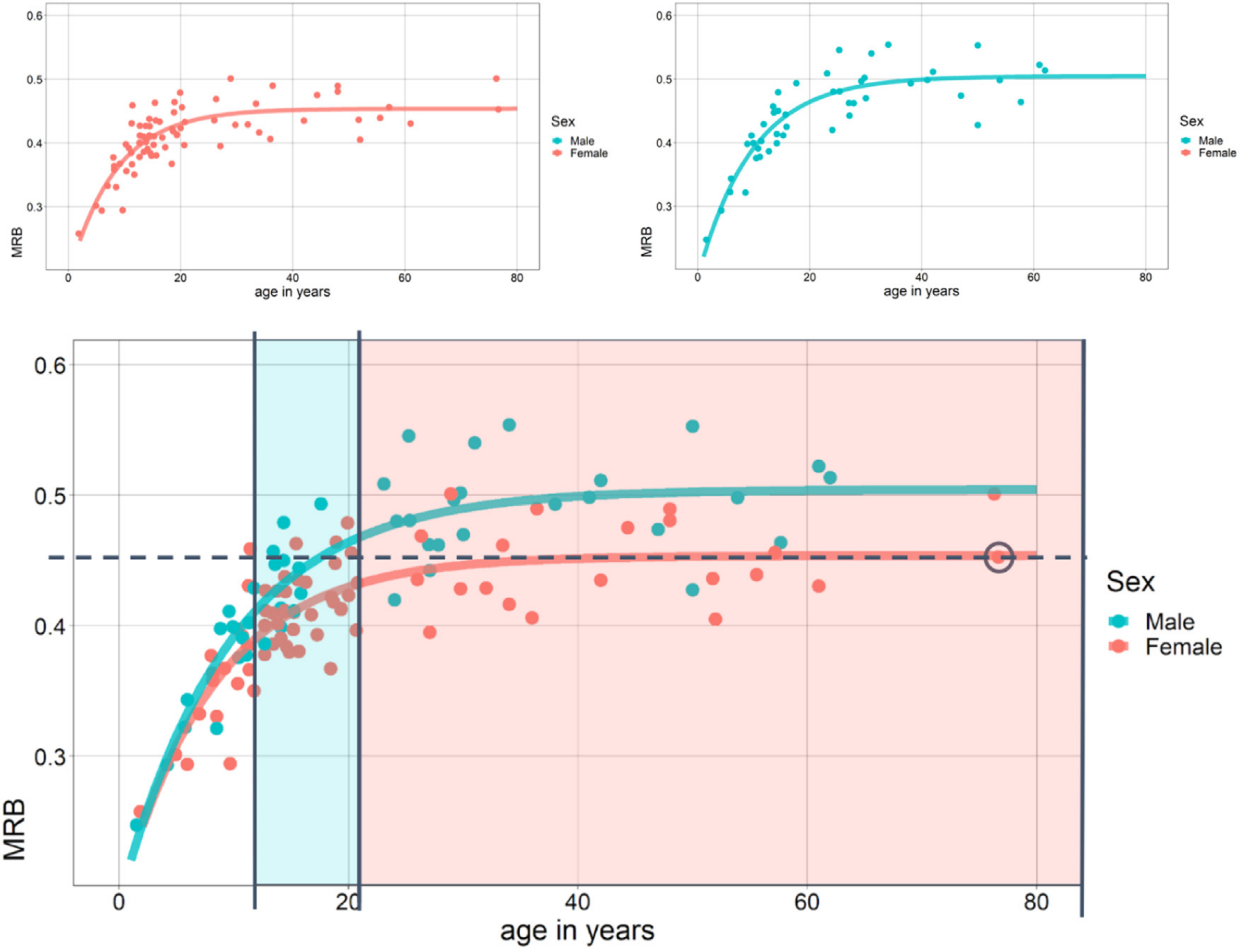
Here individuals’ mean ridge breadth (MRB) is plotted against their age and sex. The asymptotic best-fit lines are shown for each sex above. Below, as an example, an elderly woman (data point is circled) has an MRB of approximately 0.45 mm/ridge (the dotted line), which is essentially the expected value for women older than 20 years of age (pink region). However, this MRB value is also similar to the expected value for male adolescents (blue region). Without more information, the sex of a potter with such an observed MRB is equivocal. Data from Králík & Novotný [5], asymptote regression lines from this study.

The modern fingerprint dataset published by Králík & Novotný [5] demonstrates this precise relationship between age, sex, and MRB by including participants from a wide range of ages. As expected (and shown in Fig. 2), individuals’ MRB measurements can be accurately modeled by an asymptotic function when both age and sex are already known; information inaccessible to the archaeologist. The crux of this inferential ambiguity is highlighted when considering the fingerprints of hypothetical early adolescent males and older adult women. The example in Fig. 2 demonstrates how the MRB of a woman in her 70s (or any age from 40 upwards, where the average MRB is 0.45 mm/ridge) would be indistinguishable from a male teenager.

#### Problem 2: Inter-assemblage variation caused by biological and technological differences

The second limitation is that of inter-assemblage variation. Average adult epidermal ridge density measurements vary widely among different contemporary populations, according to a combination of genetic and life history factors. (Although to our knowledge, there is no evidence that this fingerprint variation represents anything separate from population differences in height and size). The coefficient of variation between average adult male MRB values is around 15% in modern studies (see [13]: Table 1 for an overview of these values). In archaeological contexts where malnutrition is endemic, we can expect life history factors to decrease MRB values by some unknown factor (see [21,22]), with the consequence that simple genetic proximity is not enough to determine a proper modern reference population. For example, if an ancient population has a high prevalence of disease and malnutrition that stunts growth, these adults would have smaller MRB values than their healthy, modern counterparts (even if they were genetically similar). Additionally, there is substantial intermanual and interdigital variability even within a single individual (Králík et al. [23]).

**Table 1:** Rough categorization system of assemblages based on their distribution of mean ridge breadth values (after extreme outliers are removed).

Low CV <0.09	Intermediate CV ∼0.09 - 0.11	High CV >0.11
Type 1:	Type 2:	Type 3:
Solely adult men or solely adult women	Adult men and women together OR adult women with adolescents (and possibly preadolescents)	Growing children OR children with adults of one or both sexes or adolescents

Furthermore, shrinkage patterns based on clay type will also affect the observed archaeological fingerprint measurements. Due to differences in clay fabric and inclusions, along with variation in firing techniques, this shrinkage can vary from around 2-12%, according to data published by clay-producing companies.

**Fig. 3:**
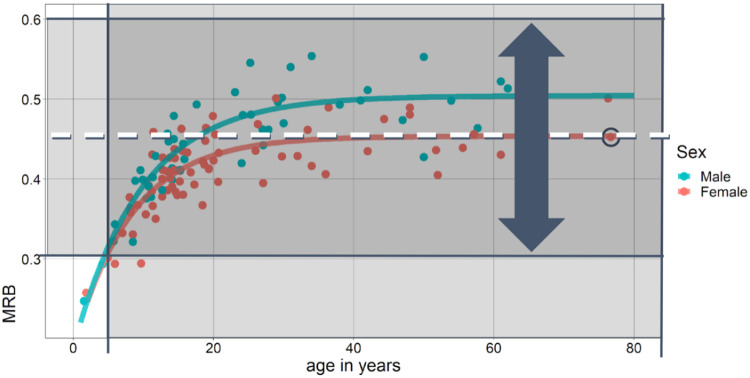
Given the observed variation between human populations and clay shrinkage rates, the observed mean ridge breadth (MRB) impression of 0.45 mm/ridge (dotted line) from the elderly woman mentioned earlier (circled data point) could have represented an MRB between ∼0.3 and 0.6 mm/ridge in another population (shaded region between the two horizontal lines). Provided this range of MRB values, the potter's age could have realistically been anything greater than four years old. Data from Králík & Novotný [5], asymptote regression lines from this study.

We can use Králík & Novotný’s dataset once again to illustrate the effects of these limitations. Considering both the shrinkage from clay and the differences between human populations, the data point we mentioned earlier (with a MRB value of 0.45 mm/ridge) could be produced by a finger with any value from approximately 0.3 to 0.6 mm/ridge. This potter could thus be male or female, or of any age above four years old (Fig. 3). In other words, the MRB of a single fingerprint can tell researchers very little about the age and sex of the potter who made the impression. Even with larger sample sizes, these limitations remain if the fingerprints are analyzed independently.

### A statistics-oriented solution

One aspect that both limitations share is the near impossibility of determining any accurate demographic information from measurements of a single archaeologically recovered fingerprint. With an isolated measurement of average ridge-furrow pair width, inter-population variation and ceramic shrinkage cannot be assessed, and even if it could be assumed, age- and sex-effects on this measurement cannot be accurately disentangled. This observation implies that the only way to derive accurate demographic information from an archaeologically recovered ceramic assemblage is by using features of the *distribution* of measurements from the overall assemblage. For instance, suppose a preserved fingerprint resembles those of contemporary 10-year-olds. It may have been produced by a 10-year-old child; however, it may also have been produced by an adult from a physically smaller population and subject to shrinkage. Focusing solely on the fingerprint in isolation we cannot determine the identity of the potter; however, considering the overall assemblage can provide additional insights. For instance, suppose the same assemblage includes many other fingerprints, some of which are as large as contemporary adult male fingerprints. Given this, we can conclude with a reasonable degree of confidence that shrinkage is modest and so the initial fingerprint likely does come from a child. Thus, pooling information across an assemblage allows more confident conclusions about each individual print and the potters that made them. Here we propose a three-step process that marshals several statistical techniques to categorize and analyze the demographic distribution underlying an archaeological ceramic assemblage. These analyses again rely on the data from modern ceramics collected by Králík & Novotný [5] using the MRB methodology, supplemented by other modern MRB datasets like David [17] where relevant.

#### Step 1: Characterize the distribution

Here we propose a basic first step that researchers can take before analyzing an ancient dermatoglyphic dataset. To perform an initial examination of the data and reduce the likelihood of testing wildly implausible hypotheses, the distribution of individuals’ MRB values can be used to very roughly classify the assemblage among several types. Specifically, we can expect that populations of solely adult male or solely adult female potters will have the least variation in MRB values. Populations with growing children or children and adults will have the largest variation in MRB values. Between these extremes, intermediate levels of variation could be produced by populations of adult men and women together, adult women with adolescents and pre-adolescents, or some similar mixture.

For this purpose, we suggest using the coefficient of variation (CV) of the dataset to characterize it. The CV describes how wide the distribution is (normalized to the sample's mean), giving us a very rough approximation of its demographic composition. Using observed values for mean and standard deviation among living populations given by (Králík & Novotný [5]: 10), the following approximate conclusions can be drawn from an archaeological sample's coefficient of variation (Table 1). This step also helps determine whether the Bayesian model (discussed below) should be run in its single sex version (for Type 1 samples) or two-sex version (for Type 2 and 3 samples). It should be emphasized, however, that this is a relatively coarse-grained classification, especially when the CV is close to the boundaries between types. It is also important to note that CV is affected by the number of ridges averaged for each individual print, particularly when low numbers of ridges are averaged in individual prints. It is for this reason that we do not advise including measurements from prints with fewer than six ridges averaged, and it is preferable when only prints in which at least 10 ridges could be averaged are included. Of course, there is a trade-off between analyzing a higher sample size of prints and analyzing only more complete prints.

#### Step 2: Scale MRB values to a reference dataset

**Fig. 4:**
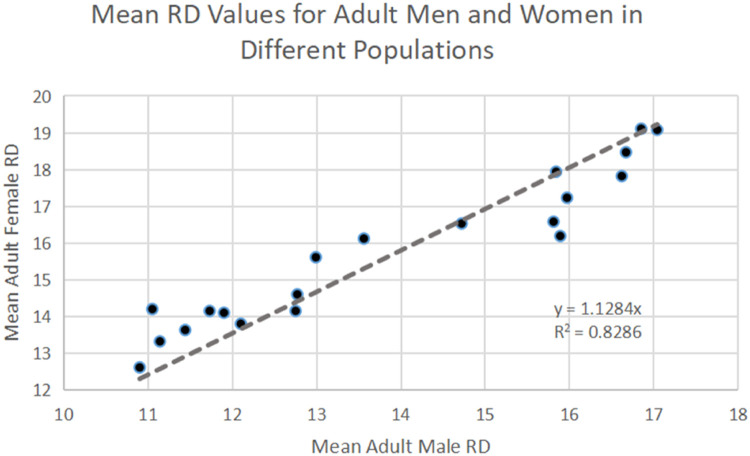
There is a strong linear relationship between adult male and adult female epidermal ridge density estimates. Here, the adult average ridge density (RD) values from different populations are plotted by sex. Data from Fowler et al. [13].

Although there is variation in ceramic fingerprint impressions caused by biological—genetic and life history differences between populations—and technological factors—clay composition and firing techniques—there are potential methods to mitigate this uncertainty. Despite the wide variation between worldwide populations, the relationship between average epidermal ridge densities from adult men and adult women remains constant across populations (Fig. 4). Given this linear relationship, if the average adult density of either sex is known then the average adult value of the other sex can be easily calculated. In other words, populations can be made analogous to another by use of an appropriate scaling factor: if we multiply the MRB values of one population by a factor that makes the average MRB values from adult men in each population similar, then the average values from adult women will also be made similar. However, it should be noted that this approach will not address all sources of statistical noise. For example, predictable interdigital variation in MRB values are not accounted for. Such variation is unlikely to introduce bias though and will merely decrease the model's precision.

Additionally, differences in assemblages in clay firing and shrinkage also change fingerprint ridge densities by a fixed, scalar value. Thus, we can use one scaling factor—subsuming both the biological and technological differences between the two consistent assemblages—to make two populations of archaeological prints comparable, without major effects on the interpretation of demographic makeup. Important for our analysis, this allows us to compare ancient archaeological samples to the modern Králík & Novotný [5] data, employed here as a reference population. However, using a scaling factor requires that we have an estimate of either sex's adult MRB value from both populations; we need to have a point of alignment around which we can adjust the scaling factor.

**Fig. 5:**
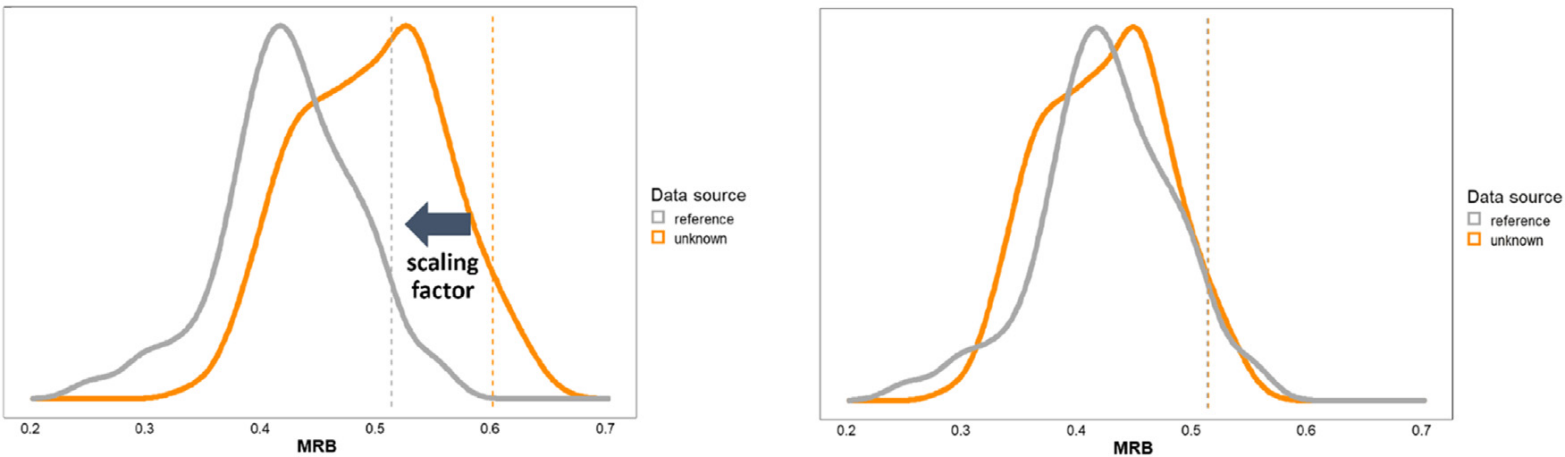
Graphical representation of using scaling factors to make datasets comparable. On the left, the mean ridge breadth (MRB) distributions from an archaeological assemblage being analyzed (in orange) and a modern reference dataset (gray) are shown. (The reference dataset is from Králík & Novotný [5] and the “unknown” dataset was collected from Hama, Syria (see [11])). Unless researchers posit that the extremely large MRB values from the unknown assemblage represent some race of ancient giant men, the mismatch between these distributions indicates likely differences in clay type or firing techniques, etc. The vertical dotted lines represent the 95^th^ percentiles in MRB values from each dataset; these are assumed to be adult male fingerprints. When multiplying the MRB values in the archaeological dataset by the correct scaling factor, the chosen percentiles become aligned across assemblages, shown on the right. Thus, MRB values in the archaeological dataset are now analogous to the reference distribution and their demographic information can be more easily interpreted.

Since the demographic make-up of the ancient fingerprint producers is uncertain, we cannot objectively estimate the adult MRB of either sex without making assumptions. However, in many scenarios it is reasonable to assume that *some* adult males participated in the production of the archaeological assemblage and, because adult men are at one extreme of the MRB range, we can rely on this to scale measured MRB values. (Due to this assumption, a different scaling method should be used where analogous ethnographic contexts or relevant written sources make it unlikely that adult men were engaged in pottery making.) Because adult males have the largest MRB values, we would expect the upper range of values to represent adult males in any dataset where they are present. Thus, we choose adult male MRB values to be this alignment point in our methods. Because we do not want to assume a large proportion of adult males, or to be misled by outlying fingerprints, we determined the scaling factor by comparing the 95^th^ percentile of MRB values from our archaeological samples with the corresponding percentile from Králík & Novotný’s [5] sample (Fig. 5). In other words, we assume that the largest 5% of MRB values in our datasets represent adult males. We also reran our analyses using the 97.5^th^ percentiles as alignment points, but in all our archaeological populations, the choice of percentile between these two options did not significantly affect our results.

To make the determination of this scaling factor more accurate, Králík & Novotný’s sample values should be filtered according to the type of sample determined for the archaeological assemblage in step 1. For type 1 assemblages, the Králík & Novotný values should be filtered to include only males aged 15 and above. (If other external data suggests a likely female-only or children-only dataset, our model could be modified to accommodate this.) For type 2 assemblages, the Králík and Novotný values should be filtered to include males and females aged 15 and above, and for type 3 assemblages, the entire Králík & Novotný sample should be used.

Note that for these methods to be valid, the data to be analyzed should be taken from a relatively homogenous archaeological assemblage. Ceramics with production contexts from very distant periods or geographic locales could be produced by potters with different genetics, life histories, and these ceramics could be made from drastically different clay fabrics. However, this does not necessarily preclude analysis across multiple periods or artifact types, but every time a separate scaling factor is calculated for a subset of the assemblage, the researcher assumes that there are at least some adult males involved with the production of that subset. Therefore, there is a balance between lumping together too many disparate assemblages and splitting an assemblage too finely, and this balance relies upon the researcher's expert discretion.

For the first multi-period archaeological sites tested with this model (see [11]), we used a common scaling factor across each entire site, rather than creating multiple factors for all ceramic types and periods. Determining a scalar value for the entire site meant that we only had to assume adult males were involved in the production of *some* ceramic objects, in *some* periods under study, rather than repeatedly making this assumption independently for the production of all types of ceramic objects in all periods of the site. This decision was made because some clay object types appear to be made by non-professionals and because we believed that variation in adult stature and clay sources at the sites were minimal (see [11] for more details).

#### A brief introduction to Bayesian mixture models

In a Bayesian mixture model, the observed data is assumed to be generated from a mixture distribution—two or more combined distributions—where each component in the mixture represents a distinct subpopulation or cluster. In this case, those distributions represent different demographic groups. The goal is to estimate the unknown parameters of the mixture model, including the proportions and the parameters (age and sex) of each component distribution. Fingerprint MRB measurements are thus a prime candidate scenario for analysis: archaeologists want to discern the subgroups (and their relevant demographic characteristics) present from an unknown mixture of MRB measurements.

Bayesian mixture models also have several key advantages over traditional statistical techniques. Firstly, Bayesian analysis explicitly incorporates prior knowledge into the modelling process. This allows archaeologists to use existing data from similar populations to inform the model itself. Secondly, Bayesian mixture models provide a framework for estimating uncertainty by producing posterior distributions of the parameters. Essentially, the degree of uncertainty itself is propagated through the model to obtain point estimates (e.g., posterior mean or median) as well as corresponding interval estimates (e.g., credible intervals). Finally, Bayesian mixture models offer flexibility in modeling complex data patterns. They can be easily altered or customized to address different situations as researchers see fit. We fully expect that our model represents an initial starting point that will be improved and adapted by future researchers.

#### Step 3: Apply the Bayesian model

After scaling the archaeological dataset to be comparable with modern reference data, the third step is to run the Bayesian model that serves as the centerpiece of this newly proposed methodology. To pool information across fingerprints and estimate both the age distributions and sex distributions of these potters simultaneously, we created a Bayesian mixture model using Markov Chain Monte Carlo methods in JAGS executed from R with the package ‘rjags’. Estimates were based on 10 chains per dataset, ran until the effective sample size of all parameters was over 3000 and the upper confidence interval of the Gelman-Rubin convergence diagnostic was less than or equal to 1.01.

**Fig. 6:**
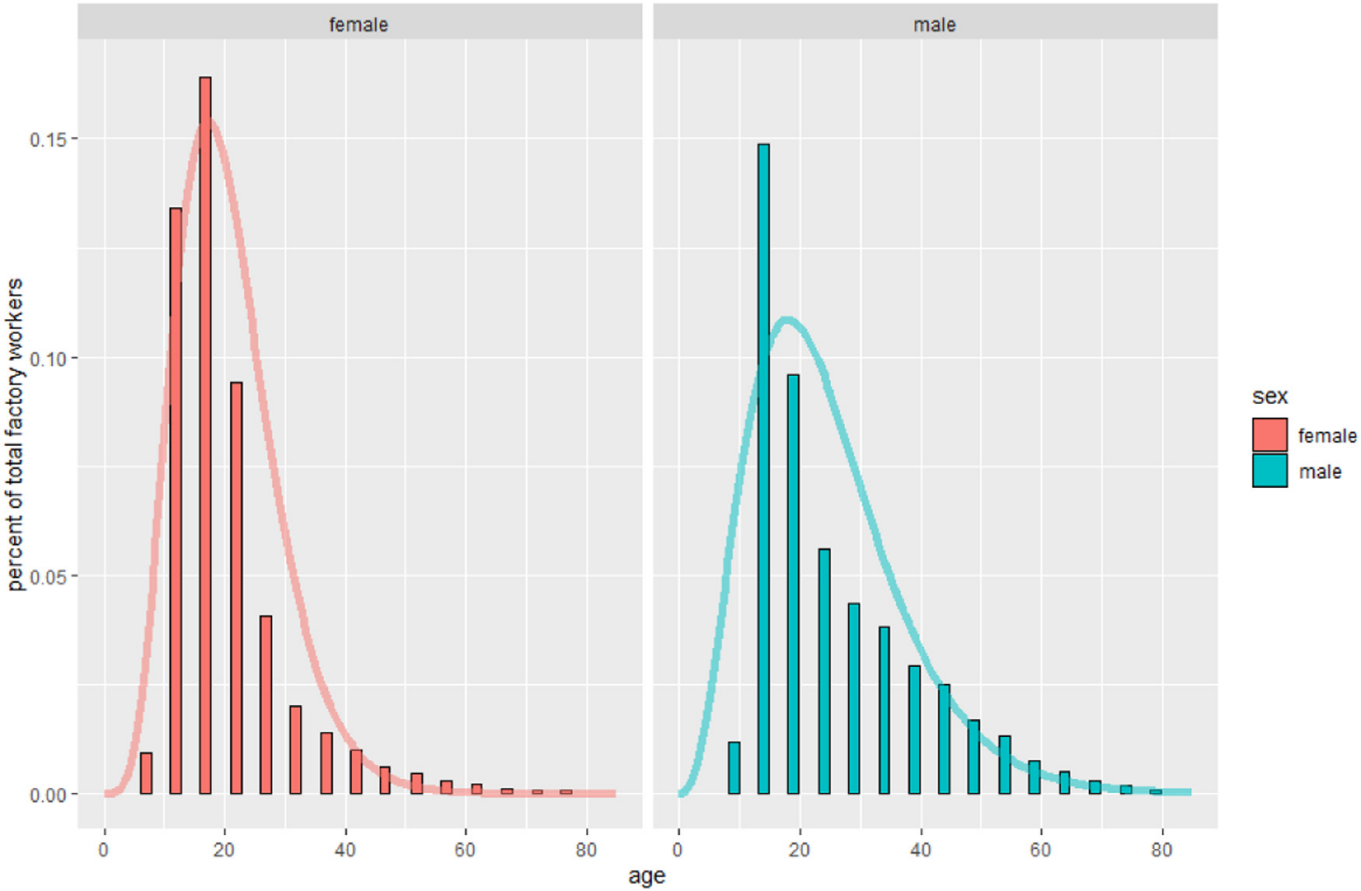
Age and sex distribution of British factory employees in 1833, fitted with gamma distributions. Note the positive skew. The slight underestimation of adolescent male factory workers is likely due to the model not understanding that very young children (e.g. 4-year-olds) cannot work in factories, so it does not understand why they are missing. Data from Mitchell [24].

**Fig. 7:**
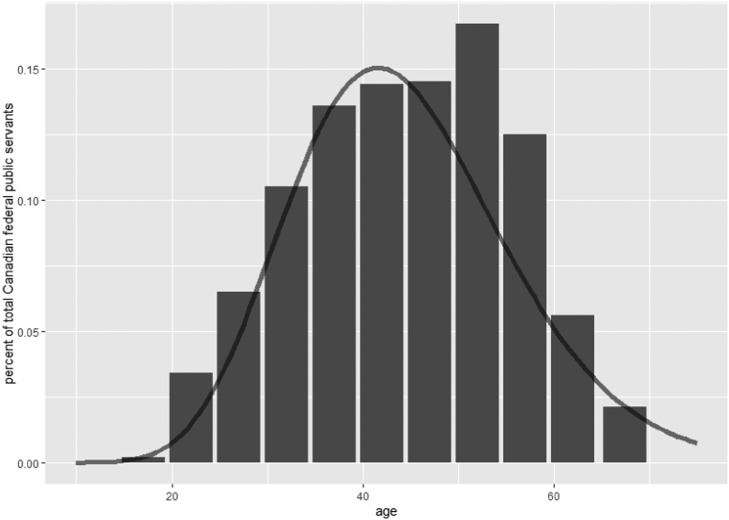
Age profile of the Canadian federal public service employees in 2016, with a gamma distribution fit to this data (black line). Note the relatively bell-shaped curve of the gamma distribution. Data from the [25].

Our model makes several key assumptions. Firstly, our model assumes an asymptotic relationship between age and MRB for each sex, based on the experimental data collected by Králík & Novotný [5]. Specifically, the model assumes MRB grows asymptotically with age, with growth defined by the initial MRB value (at age 0), its rate of growth and its final plateau during old age. Secondly, it assumes that the age distribution of the fingerprint-generating potters can be described with a gamma distribution for each sex. Gamma distributions are used to model continuous variables that are always positive, and the resulting distributions range from “normal-looking” to positively skewed. They are very flexible and can easily represent various workforce demographies, as seen in Figs. 6 and 7; however, they cannot produce negatively skewed, or multimodal distributions. Lastly, the model can optionally assume that the fingerprint with the largest MRB measurement in the given dataset represents an adult man and will explicitly label it as such. Although this last assumption is not required, it can aid in model identifiability, and given our assumptions about scaling between datasets discussed earlier, researchers may feel that this imposes only a minor additional constraint.

Whereas an age distribution is a gamma distribution that describes the relative frequencies of different ages, the “sex distribution” is a single a number ranging from 0 to 1 which corresponds to the proportion of the population that is male (i.e., 0 corresponds to all women, 1 corresponds to all men, 0.5 is an equal mix of men and women, and so on).

Bayesian analyses require mathematical specification of our beliefs prior to collecting relevant data. For the sex distribution, we used a flat (or uninformative) prior such that all possible values of the sex distribution were, *a priori*, equally likely. The age distributions of male potters and female potters are described with gamma distributions for which we provided priors concerning the mean age and the standard deviation of age. Weakly informative priors were used, such that the model assumed the mean age of potters of either sex was most likely around 20 but mean ages between 5 and 45 were all plausible. While the model favored small values for the standard deviation in the age of potters, anything up to 35 was plausible. These starting beliefs are deliberately vague, and the model updates its estimates based on the data presented. The priors that describe the relationship between age, sex, and MRB (*Asym, R_0_*, and *lrc*) were drawn from the asymptotic regression run using Králík & Novotný’s data [5]. The precision for these prior distributions was purposefully left large to give the model flexibility.

The mathematical structure of the model is as follows, with the parameters explained in Table 2:

**Table 2:** Parameters and their interpretations in the core Bayesian mixture model.

Parameter	Interpretation
*MRB_i_*	observed mean ridge breadth (MRB) of individual fingerprint *i* (likelihood function)
*u_i_*	the expected MRB value for individual *i* (likelihood function)
*σ*	standard deviation around the expected MRB values (prior)
*age_i_*	estimated age of individual *i* (likelihood function)
*sex_i_*	estimated sex of individual *i*; 1 = female, 2 = male (likelihood function)
*probMale*	estimated sex ratio of individuals in the dataset; 0 = all female, 1 = all male (prior)
*Asym*_1:2_	the horizontal asymptote in the MRB growth equation for each sex; this is the expected MRB value for an elderly adult (prior)
*R_0_*_1:2_	the response when age is zero for each sex; this is the hypothetical MRB of newly born infants (prior)
*lrc*_1:2_	the natural logarithm of the rate constant for each sex; this determines how quickly MRB increases with age (prior)
*μage*_1:2_*, σage*_1:2_	hyperparameters representing the mean age and standard deviation of ages for each sex
*α*_1:2_*, β*_1:2_	the shape and rate formulations of the above hyperparameters



Essentially, the model works by simultaneously estimating population level parameters (i.e., an age distribution for each sex and the relative frequency of the two sexes) and individual level parameters (i.e., the age and sex of each specific fingerprint). These inferences inform each other; for instance, the model's conclusions about each fingerprint are informed by what it learns about the population of fingerprints, but what it concludes about the population is also informed by what it learns about each specific print. Estimates are generated by using the relationship between age, sex, and MRB found from Králík & Novotný’s [5] data: the model's hypothetical age distributions are transformed into expected MRB distributions, which can be compared with the observed dataset, and values are considered plausible if they reduce the discrepancy between expected and observed MRB values. However, the model's output includes its uncertainty, so conclusions about all parameters are described with probability distributions over plausible values, as opposed to single “most likely” values (often called “point estimates”). Thus, the results of the analysis are probability distributions for each data point's age and sex—encoding the inherent uncertainty in these measures—as well as for the parameters describing the age and sex distributions of the population of potters.

Note that before the scaled MRB values are passed to the model, the data can and should be divided into self-consistent categories that the researcher assumes were made by similar populations and analyzed separately. For example, when analyzing the clay objects from Hama, Syria, we divided the assemblage into full sized pottery, miniature vessels, and figurines because we were not comfortable assuming the groups making these categories all had a uniform demographic profile. After employing a site-wide scaling factor, we ran the Bayesian model separately for each of these categories [11].

One advantage of the Bayesian approach is that prior knowledge of the underlying processes is explicitly incorporated into the model. This allows us to easily and flexibly adjust the model based on insights from other researchers. For example, at one site we analyzed (see [11]), our model indicated extreme differences between the ages of male and female potters. Unless given a strong reason otherwise, we believe that the models should generally allow for the presence of both men and women potters, given that the intent of these models is to estimate the demographics of these potters and the presence of such subgroups. However, in this example, the model suggested fingerprints were made from a population of older adult men and a distinct population of prepubescent female children. Given this extreme result and our awareness that young male and female children are indistinguishable by MRB alone, we further examined the dataset by testing several more refined variants of the model representing alternative hypotheses.

Another advantage of Bayesian modelling is the ease in altering model parameters. In the above example, we wanted to investigate the specific mixture components of the model (the likely demographic groups). However, when choosing from candidate models of similar predictive or explanatory power, the simplest model is most likely to be the best choice. We could posit fingerprint models that estimate parameters for *many* different subgroups at once, but the complexity of these models expands exponentially when adding extra mixture components. Such models quickly lose the ability to make useful estimations of demographics. However, in Bayesian model selection using Bayes factors, our preference for simpler models with fewer parameters is built-in.

When analyzing the data from Hama, Syria, we tested several similar models. One variant of the model we considered is the “two sexes, one age distribution” model. It is similar to the standard model described above, yet both sexes share a single age distribution. Instead of allowing each sex to have its own, independent age structure, it assumes men and women enter and leave the fingerprint-generating workforce at similar rates across their lifespans. Another variant we generated is referred to as the “one sex, two age distributions” model. Here, all individuals are assumed to be a single sex (male), but we allow two distinct age groups among the potters. We pitted these models against the original by using a transcendental “supermodel” that combines the various models being compared and fits them simultaneously. This approach estimates the relative likelihood of the different models and can be used to assess which model best explains the data. It essentially calculates Bayes factors for each pair of models. In this example, the “one sex, two age distributions” model was favored, strengthening the interpretation that two distinct age classes were involved in pottery production. Sanders et al., [11] interpreted this result in the context of the larger region: the authors demonstrate that nearby sites responded to increased social coordination and wider exchange networks in different ways. Sites began to use either child labor or single-sex professionals (and in the case of Hama, perhaps both) as strategies for selecting and training workers to increase productivity.

### Comparing our model's performance to existing methods

To evaluate the performance of our model, we compared it against other methods in the literature over a range of simulated demographic scenarios. In these simulated scenarios, the relationships between age, sex, and MRB were the based on the asymptotic regression built from Králík & Novotný’s [5] experimental data. Additionally, realistic noise was added to the simulated MRB values based on the root-mean-square error of the residuals from this regression. Age distributions for each sex were independently gamma distributed.

We generated 540 simulated datasets, varying the simulated sample size, the proportion of females, and the mean age and standard deviation of both men and women independently. Sample sizes were either 50, 100, or 200; the proportion of females was either 10, 30, 50, 70, or 90%; mean ages for each sex were either 15, 20, or 30 years old; and standard deviations of ages for each sex were either two or eight years.

The demographic models were taken from previous literature. We refer to our model as “bayes” when listing models. We used several of the linear models used in Králík & Novotný [5] comparing age and MRB: a model called “PM1” based off their data; their “KAmod” model, a modified version of Kamp et al.’s [7]; as well as both Loesch and Czyżewska's [26] model (“LC”) and the updated version of this model called “LCmod.” We also included “KAmod2,” which is Fowler et al.’s [13] and [12] modification to Kamp et al.’s [7] model. Lastly, we added two random models as baselines: a randomly guessing model (called “random”) that would randomly choose a sex and any age from five to sixty as well as an educated guessing model (“random2”), which would randomly choose a sex and would randomly choose ages from a distribution centered around 18 years old with a standard deviation of five years.

Using all the models, we compared their ability to identify the age distributions of our simulated potters. Additionally, for the models that infer both the age *and* sex of potters (our Bayesian models, KAmod2, and the random models), we also compare their ability to discriminate between simulated male and female potters. In Fowler et al. [13] and [12], data points are marked as male when the ridge density (RD) was below 13 ridges/25mm^2^, but our dataset did not include RD measurements. However, because RD and MRB are essentially inverses of one another with MRB explaining 94% of the variance in RD, we safely calculated RD measurements from our simulated MRB datasets. To boost the potential accuracy of Fowler et al.’s [12] KAmod2 model, we rounded the calculated fractional RD measurements belonging to women *up* to the nearest integer and rounded *down* those of men. Further aiding this model, we found that the 13 ridges/25mm^2^ cutoff originally calculated by Gungadin (2007) [10] and used by Fowler et al. [12] did not accurately describe Králík & Novotný’s [5] dataset, so we used a logistic regression classifier to find the optimal RD threshold for sex determination (14.9 ridges/25mm^2^) and used this in KAmod2.

In models that infer both sex and age (our Bayesian models, the random guessing models, and KAmod2), we calculated the inferred total mean age, the percentage of females, the mean ages for each sex, as well as the total percentages of children, adolescents, and adults for each sex. The differences between these estimates and those from the actual simulated data were then compared. To also include the models that only inferred age, we reran the analyses calculating only mean age and the percentages of children, adolescents, and adults, irrespective of sex.

#### Model comparison results

**Fig. 8:**
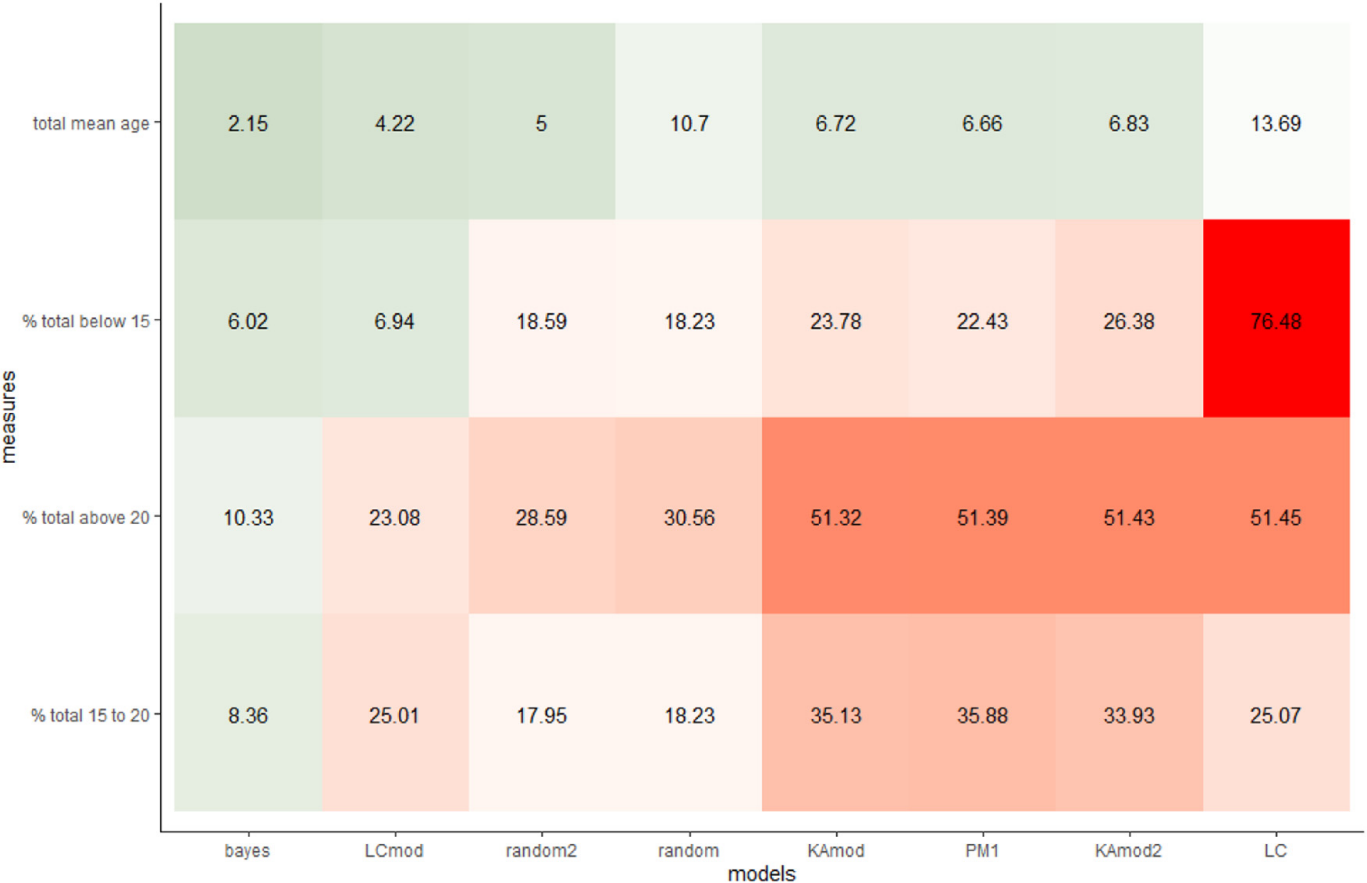
Comparing model accuracy for age-based demographic metrics across 540 simulated datasets. The cell values represent the average absolute difference between the specific model's estimate of a metric and the actual value generated by the simulation: lower values imply higher accuracy. Note, the randomly guessing model (“random”) and the educated guessing model (“random2”) outperform most models.

**Fig. 9:**
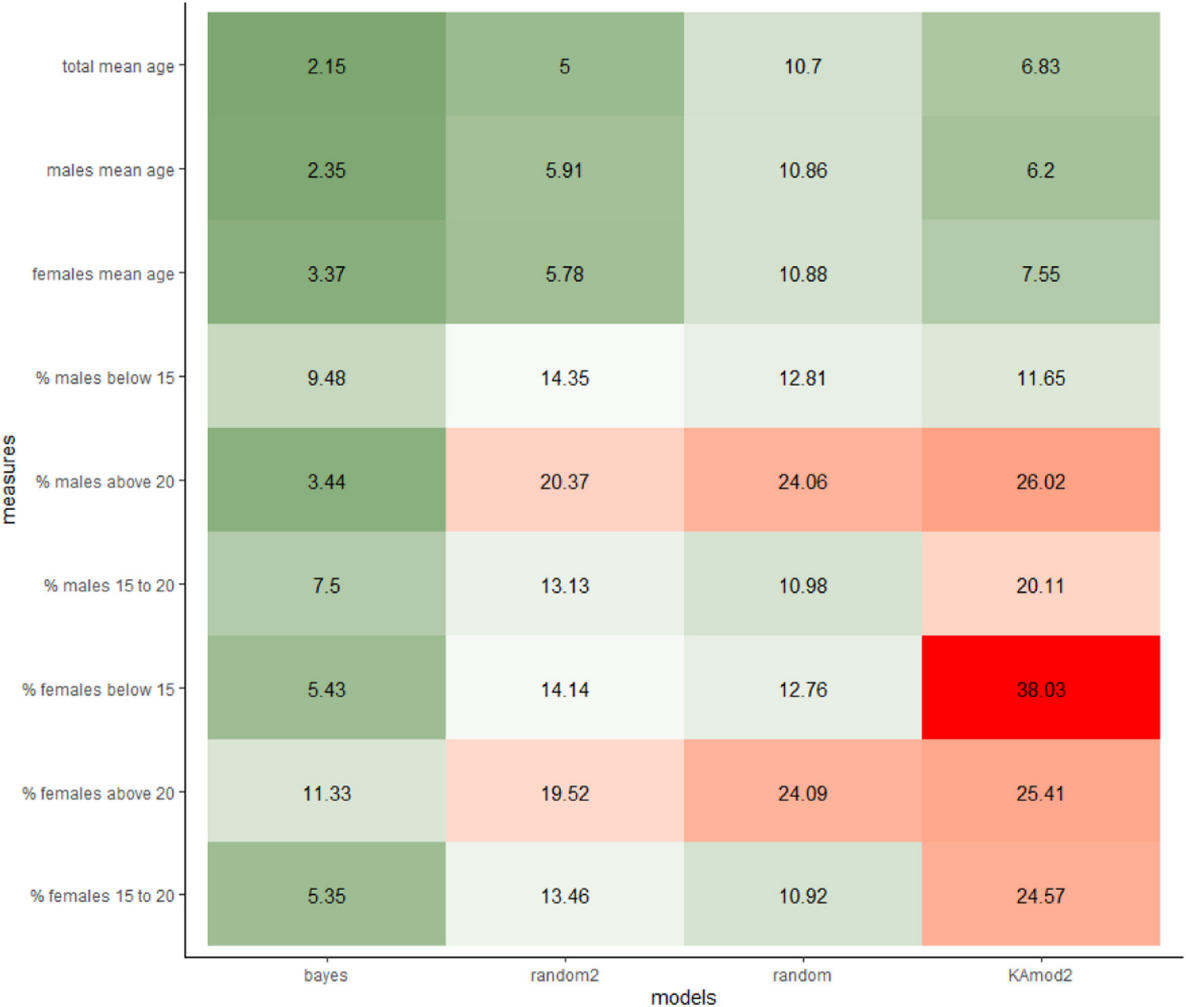
Comparing model accuracy for age- and sex-based demographic metrics across 540 simulated datasets. The cell values represent the average absolute difference between the specific model's estimate of a metric and the actual value generated by the simulation: lower values and darker green cells imply higher accuracy. Note, the randomly guessing model (“random”) and the educated guessing model (“random2”) outperform the “KAmod2” model.

Our Bayesian model outperformed the other models when inferring all age-related estimates and when inferring all simultaneous age- and sex-related estimates (Figs. 8 and 9). Importantly, when comparing accuracy scores averaged across the different estimates, the random guessing models outperformed all other models except our Bayesian model and Králík & Novotný’s [5] updated version of Loesch and Czyżewska's [26] model. These results highlight the impossibility of accurately inferring potter demographics when analyzing prints individually. However, given the lack of comprehensive, modern fingerprint impression datasets that include both sexes and the gamut of human ages, both our model and the simulated data were based on the same data from Králík & Novotný [5]. Our model evaluation process is thus at some risk of overfitting, at least until further experimental datasets are collected.

### Summary and conclusion

Although fingerprint impressions on ceramic artifacts have the potential to tell us about the ancient potters that produced them, past attempts to use epidermal ridge density as a demographic predictor have been systematically flawed. Both age and sex cannot be estimated from a single data point; age and ridge density do not have a linear relationship; and variation between assemblages renders comparisons essentially futile. It is only by pooling information within an assemblage and making several simple assumptions that demographic inference is possible. In our Bayesian mixture model approach, we assume that at least some small percentage of the fingerprints come from adult men, that the distribution of ages in a workforce is roughly gamma distributed, and that the relationships between age, sex, and epidermal ridge density follow an approximately asymptotic shape (as seen in the data). This allows us to estimate potter demographics more accurately than previous methods *and* directly quantify our uncertainty in these estimates.

Although there are inherent limitations in any approach, we believe our method of using mean ridge breadth values to estimate potter demographics is superior to existing approaches. For example, although our method relies on a sample of fingerprints to estimate any demographic data, it still outperformed existing methods when only 50 MRB values were used (the smallest sample size we tested). Biases in the data itself also impact these methods, though such biases will have similar effects on all existing methods. Lastly, although our model makes several key assumptions, the Bayesian framework makes these few assumptions clear, realistic, and mathematically explicit. No other current models can realistically account for both age and sex estimation.

The field of archaeological dermatoglyphics is still in its infancy. Moving forward, more modern experimental data is sorely needed, especially research comparing impressions across distinct geographic populations and across distinct clay firing techniques. Additionally, more independent predictors of demography should be explored: other ridge characteristics like whorls, bifurcations, and spurs may be able to aid in inference. Hierarchical analysis could also be fruitful avenue of research: multiple fingerprints from a single artifact or multiple estimates from a single fingerprint could be analyzed in ways to add statistical power. Regardless of what future research finds, a Bayesian modelling approach will continue to represent our best chance to incorporate these findings into new analyses. These models force us to make our assumptions explicit, and they can easily be modified to fit the specific needs and goals of every research team.

## Declaration of Competing Interest

The authors declare that they have no known competing financial interests or personal relationships that could have appeared to influence the work reported in this paper.

## Data Availability

Data will be made available on request.
